# Pandemic responses: Planning to neutralize SARS-CoV-2 and prepare for future outbreaks

**DOI:** 10.1371/journal.pmed.1003123

**Published:** 2020-04-28

**Authors:** The PLOS Medicine Editors

**Affiliations:** Public Library of Science, San Francisco, California, United States of America and Cambridge, United Kingdom

## Abstract

The PLOS Medicine Editors discuss the current SARS-CoV-2 outbreak and implications for global health.

In February, in the course of discussing possible challenges to international health plans for the 2020s, we noted in passing that “a new coronavirus outbreak [was] emerging in Asia at the time of writing” [1]. Vividly illustrating the pace at which an infectious disease outbreak can progress among a highly interlinked and susceptible global population, in the intervening few weeks a pandemic has not only taken hold but already reached virtually every country. As of 30 March, almost 700,000 cases of COVID-19 disease had been confirmed worldwide, with the severity of disease highlighted by the more than 33,000 deaths reported among those infected [2]. The severe and growing consequences of the pandemic are likely to have transformational effects on people, societies and health systems worldwide, and highlight the degree of interconnection of individuals and countries—calling for intensified and coordinated preparation and action in the future.

The new pathogen, Severe Acute Respiratory Syndrome Coronavirus 2 (SARS-CoV-2), is closely related to other coronaviruses, including the causative agent of the more limited SARS outbreak that occurred principally in China and Hong Kong during 2002–04 [3]. The new virus emerged in a cluster of pneumonia cases detected in Wuhan, central China in December 2019. The virus had been identified in early January and, by the time that cases had been documented in several other countries, on 20 January, the risk of a serious disease outbreak was already clear [4]. The shocking speed with which SARS-CoV-2 has spread indicates not only the vulnerability of people and communities to threats posed by infectious diseases, but the challenges of mounting swift and effective responses. As has been widely reported, the city of Wuhan and its 11 million people were “locked down” in late January, although this dramatic action was evidently too late to limit the infection’s breakneck global expansion [5].

Much remains to be learned about SARS-CoV-2 and its effects. We can surmise that the virus is readily transmitted from person to person, in a similar manner to other respiratory pathogens, before symptoms are pronounced. Disease severity seems to vary markedly, with serious respiratory diseases, including pneumonia, developing in a proportion of patients, often elderly people and those with comorbidities. It is evident from the scholarly publications so far that available treatments, including some antiviral drugs, are being employed in patients; although various clinical studies have been done and reported impressively quickly, specific drug effects are difficult to assess [6]. PLOS and other publishers have played a part in encouraging early communication of research findings, and the relevant literature is growing. It is to be hoped that clinical and supportive care is developed to a point that the uncertain but apparently substantial death toll in infected people—estimated at 0.25–3% [7]—can be mitigated in settings with sufficient health provision. Development of vaccines is, unfortunately, likely to lag behind the outbreak by a year at least.

Could individual national authorities and agencies have responded better? There has been no lack of major infectious disease outbreaks in recent years, recalling SARS (2002–04), Middle East Respiratory Syndrome (MERS; 2012–19), Ebola (2013–2016 in West Africa and 2018–20 in the Democratic Republic of the Congo), Zika virus (2015–16) and others. Therefore, it has to be acknowledged that COVID-19 is, albeit disastrous, a valuable alarm call to the effect that no constituency or person can now be reliably protected from an emerging and unstudied infectious disease. It would be unwise to make specific predictions about the course and outcome of, or efforts to combat, the ongoing SARS-CoV-2 outbreak, but some initial conclusions can be made. WHO—an organization that has received harsh criticism in the past for its perceived passivity in the face of disease outbreaks—is essential and has performed creditably against the current challenge. The responses of individual national governments have received some very trenchant commentary, and to a degree this criticism can be explained by local political factors, the intense and sometimes distorting lens of news and social media, and the luxury of hindsight. Most organizations and their staff will have learnt important lessons over the course of the current outbreak, however, and there will be much debate and planning to come.

Those health workers in emergency medicine and other areas must surely feel that they are working in systems thoroughly unprepared for the current crisis, and deserve enormous credit. Looking to the design of an outbreak-ready future, early inferences would suggest that decisive and permanent changes in animal handling practices are needed to control, and as far as possible prevent, further zoonotic transmission events. Throughout the outbreak, there has been the impression that governments are unsure about or unprepared for the public health responses needed, and hesitant in communicating with their countries’ populations. Greater international cooperation seems essential in outbreak science and public health, and in actions to prevent disease movement between regions and countries; here, the resources and reputation of WHO can probably achieve more to foster coordinated thinking and action. The biggest challenge is that of uncertainty—how will a new virus move and manifest itself in different countries, how can potentially contradictory data and advice from models and researchers be reconciled and implemented, and how will governments manage conficting political, economic and health priorities? SARS-CoV-2 is an unprecedented challenge, and one in a sense created by the modern world. The impact in many low- and middle-income countries and on key population groups is yet to be judged. How people and health systems respond to the current outbreak will be key not only to planning for the unknown pathogens of the future but to maintaining a stable environment for the global community’s threatened, but hopefully not erased, health plans for the 2020s and beyond [1].
